# Evaluating Spherical Trees in the Urban Environment in Budapest (Hungary)

**DOI:** 10.3390/plants14020228

**Published:** 2025-01-15

**Authors:** Krisztina Szabó, Eszter Tőke, Attila Gergely

**Affiliations:** 1 Department of Garden and Open Space Design, Institute of Landscape Architecture, Urban Planning and Garden Art, Hungarian University of Agriculture and Life Sciences, 1118 Budapest, Hungary; 2 BKM FŐKERT, Budapest Public Utilities Private Limited Company, 1116 Budapest, Hungary; 3 Department of Landscape Protection and Reclamation, Institute of Landscape Architecture, Urban Planning and Garden Art, Hungarian University of Agriculture and Life Sciences, 1118 Budapest, Hungary; gergely.attila@uni-mate.hu

**Keywords:** urban trees, spherical crowns, canopy cover, health status, shadow projection

## Abstract

The world’s big cities, including Budapest, are becoming more crowded, with more and more people living in smaller and smaller spaces. There is an increasing demand for more green space and trees, with less vertical and less horizontal space. In addition, deteriorating environmental conditions are making it even more difficult for trees to grow and survive. Tree species in urban areas have multiple functions and high ecosystem services when in good health. Among taxa with diverse habits, sizes, crown shapes, growth vigor, longevity, urban tolerance, and canopy habit, our research aims to evaluate urban specimens of spherical species with smaller space requirements and sizes but have regular geometric crown shapes in public plantations in Budapest. In the restricted urban habitats, the city’s cadastral records include 4676 specimens with spherical crowns. Among the species examined, eight species with globular crowns (*Acer platanoides* ‘Globosum’, *Catalpa bignonioides* ‘Nana’, *Celtis occidentalis* ‘Globosa’, *Fraxinus excelsior* ‘Nana’, *Fraxinus ornus* ‘Mecsek’, *Platanus × hispanica* ‘Alphen’s Globe’, *Prunus × eminens* ‘Umbraculifera’ and *Robinia pseudoacacia* ‘Umbraculifera’) were evaluated in relation to age, health, wood type, crown size, and shade projection in order to show which species are or will be suitable in the future.

## 1. Introduction

Currently, the vast majority of people live in cities, and, by 2050, more than two-thirds of the world’s population is expected to live in cities [1]. The world’s major cities, including Budapest, are becoming increasingly crowded, with more and more people living in smaller and smaller spaces. The population is increasingly demanding green spaces, parks, and trees on the streets. Many people are building a lot, and to serve the needs of the growing urban population, higher-performance utilities such as sewers, electrical, and telecommunications lines are being installed underground or in the form of overhead lines. In the vast majority of cases, utilities are installed in the pedestrian zone along the road, where tree spaces are also found, so the vertical and horizontal space available for trees is decreasing. Vehicle traffic has also increased in recent decades, which greatly worsens the quality of urban air. Last but not least, many more parking spaces are needed, the design of which also generates competition with existing tree spaces. The areas where trees can survive are rapidly decreasing and the conditions where trees can survive are rapidly deteriorating. The increasing soil sealings only allow the survival of species with very high plasticity [2]. A contradictory situation has developed between trees and the urban environment, about which Dezső Radó said as early as 1981 [3] that “the more the city needs trees, the more they get in the way”. The greatest treasure of today’s green space maintenance is the tree space.

The crown shapes of the species and cultivars used in the city are diverse and also differ greatly in terms of city tolerance and foliage quality. The use of taxa with different habitus depends on the size of the available area and the environment, and may also differ depending on the age of the trees. The crown shape of the tree determines the volume of the crown and thus affects the ecosystem service of the tree, for example, the size of the shaded area or the quality of the shade. Nowak and Aevermann, 2019, also emphasize the importance of tree size, for example, in relation to pollution abatement [4], having a significant impact on maximizing health benefits and reducing spatial inequalities [5].

Within the framework of urban afforestation, green space maintainers—such as the Budapest Gardening Company (FŐKERT)—prefer to use tree species with a spherical habit; these species can adapt to the narrowed urban living space and satisfy the criteria that shaped trees with a regular geometric crown shape can be very well integrated into the architectural built environment. Franceschi et al., 2022, examined data on hundreds of crown shapes, volumes and shades cast by 3852 tree individuals in eight German cities [6]. In their evaluation, they observed mostly ovoid (57%) and spherical (24%) crown shapes and the largest shading areas were measured for the spherical shape, with the highest shade measured density for the ovoid shape. Among the advantages of spherical crown varieties, we can mention that they grow a regular spherical crown, grow slowly and their maximum height is 4–5 m [7,8]. The trunk height of spherical crown varieties remains constant at the size at the time of nursery production so they can also be planted under overhead power lines that are often present on streets. Their disadvantageous feature follows from the aforementioned fact that the small crown provides very little shade overall [9], and, in addition to the shade effect, the services and environmental value of almost all other ecosystem services and small crown-sized taxa are also lower compared to other, larger taxa. However, there are also significant differences between individual spherical tree taxa [10].

The aim of our work is to compare the dendrometric data, health status, and tree location and tree environment data of various spherical crown species in Budapest in the cadastral register of the FŐKERT. We aim to determine (1) which spherical crown taxa can actually be recommended for urban environments, which ones can live the longest, and which ones can be expected to provide the greatest ecosystem services. Our research questions also include (2) whether there are any taxa among the examined taxa that should be avoided and (3) whether there is a correlation between the type of tree location and the size of the canopy, i.e., the shadow projection, and (4) between the health status of individual taxa and the health status of the crown. The assessment of the taxa examined is based on (5) whether proximity to buildings is a determinant, and (6) which taxa are most likely to increase in urban settings over time.

Furthermore, with our findings, our important goal is to demonstrate to urban decision-makers that although some taxa have a lower initial cost, after a short time, their impact on the ecosystem is inferior to taxa that can be obtained at a slightly higher cost.

## 2. Materials and Methods

### 2.1. Field Sampling

**Figure 1:**
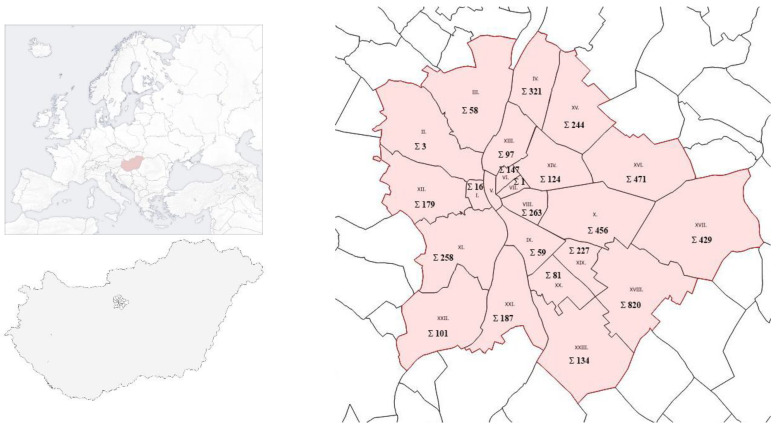
Europe, Budapest, and the spatial distribution of the spherical varieties included in the study in the districts of Budapest.

The spatial distribution of spherical species in the districts of Budapest is very variable. There are districts where small taxa appear in very small numbers (districts II and VII), and there is also a district where not a single spherical species is found (district V). The distribution of the study site and taxa by district is shown in Figure 1.

### 2.2. Evaluated Taxa with a Spherical Crown

There are around 20 different spherical varieties of the European commercial range of shaped trees. The taxa included in the study are *Acer platanoides* ‘Globosum’, *Catalpa bignonioides* ‘Nana’, *Celtis occidentalis* ‘Globosa’, *Fraxinus excelsior* ‘Nana’, *Fraxinus ornus* ‘Mecsek’, *Platanus × hispanica* ‘Alphen’s Globe’, *Prunus × eminens* ‘Umbraculifera’ and *Robinia pseudoacacia* ‘Umbraculifera’.

The American-originated *Robinia pseudoacacia* was introduced to Europe in the early 1700s [11] in 1601 with the help of the director of the Paris Botanical Garden [12] and spread rapidly due to its adaptability. It has become naturalized in many countries [13,14] and has even become invasive [15], being on the list of the 100 most dangerous invasive species [16]. It was introduced to Hungary in the early 18th century. Today, it has the largest area covered by black locusts, more than in all other European countries combined [17,18,19,20]. *Robinia pseudoacacia* is also listed as an invasive species in the Hungarian decree [21], and *Celtis occidentalis* is also listed in a decree since last year (Government Decree 282/2024. (IX.30.) [22], so it cannot be used for urban planting, except for its horticultural varieties, such as the spherical black locust, which was discovered in Austria in 1813 [23]. Its growth is slower than that of the main species, and it does not produce flowers, so it does not litter with its flowers or fruit. Its foliage remains green longer, and its leaves are smaller than those of the parent species. The rootstock (trunk) often sprouts, so it must be regularly grafted. The grafting site rots at the age of 25–30, so there is a high risk of branches breaking [24], so most individuals in serious health conditions can be observed in this taxon. Despite this, the urban application of the *Robinia pseudoacacia* ‘Umbraculifera’ is outstanding, and it is the most widespread urban ornamental tree in Budapest, but there are also a number of studies on similarly tall urban plantings [25].

The *Acer platanoides* ‘Globosum’ variety, native to Europe, was bred in Belgium in 1873. It is grafted onto a tall trunk and grows to a height of about 3–5 m with a flattened spherical crown and about the same width. Its flowering time is the same as the parent species [20]. It grows a thin but densely branched crown, the risk of branches breaking is low, and the grafting site thickens over time [24].

The popular and world-famous Hungarian variety of the *Fraxinus ornus* species, originating from Southern Europe and Asia Minor and is also native to Hungary, is ‘Mecsek’ (discovered by Ferenc Kett, a state-recognized variety since 1985) [26]. Both the parent species and the variety retain healthy foliage for a long time; therefore, among the native ash trees, the flowering ash can be considered a climate tree in addition to its good urban tolerance [10].

*Fraxinus excelsior* is a European tree species, mostly found in mountainous areas in Hungary, and prefers cooler conditions. It is a fast-growing, long-lived tree, reaching a height of 30–40 m. Its insignificant, petal-less flowers appear before the leaves emerge in April or at the same time [7]. It tolerates moderate drought and urban air well. It was a popular street tree that marked an era in the history of tree rows [8] until the appearance of the ash dieback (*Hymenoscyphus fraxineus* syn. *Chalara fraxinea*) fungal disease (although it is still planted in large numbers in Budapest). The ‘Nana’ variety was bred in France in 1805. It is a dwarf, slow-growing tree with smaller leaves than the parent species [7]. At maturity, it reaches a height of 4–6 m, with a semi-open sphere crown. Because of this characteristic, it did not become very popular. It does not grow flowers or fruit.

*Prunus × eminens* ‘Umbraculifera’ (syn. *Prunus fruticosa* ‘Globosa’) is a spherical-crown variety grafted onto a tall trunk, and it has one of the most regular spherical crowns [8]. It flowers profusely in April but does not bear fruit. It is usually grafted or budded onto *Prunus mahaleb* rootstock. It grows slowly, with an expected lifespan of 30–40 years [7]. It is widely used in Budapest, mainly as an element of architectural plantings due to its regular crown, although its crown disintegrates after a few decades.

*Catalpa bignonioides* originates from the southern states of the USA [27]. It sprouts late in spring, sheds its leaves at the first frost, has a short growing season, and is city-tolerant but not a good climate tree [10]. It usually has a stocky trunk and a spherical crown shape and is a popular ornamental tree in parks and gardens [28]. After its spectacular inflorescences, the parent species is also ornamental with its fruits after the fall of the leaves, but it is highly susceptible to powdery mildew. Its maximum vitality in urban conditions is 30–40 years [25]. The ‘Nana’ cultivar was bred in France in 1850. It develops a flattened spherical crown, is grafted onto a tall trunk, usually does not bloom, and therefore does not produce fruit. It grows to a height of 3–5 m, its leaves are smaller than those of the parent species [23], and its susceptibility to fungal diseases is similar to the parent species.

*Celtis occidentalis* is also an American species, which was introduced to Hungary in the early 19th century. It is drought-tolerant but can also tolerate waterlogged conditions and is not demanding on the soil. It produces abundantly and reproduces very well from seeds, which are also spread well by birds, which is why it is now starting to behave as an invasive species and is also on the invasive list [29,30]. The ‘Globosa’ variety is a Hungarian variety, the first spherical crown *Celtis* variety, which was bred by Zoltán Ifju in 2000. It grows quickly, the annual increment of the canes reaches 20–40 cm, and its height is 6–8 m. It tolerates pruning well. It is an excellent choice for busy roads, and it also tolerates air pollution well [31].

A well-known and frequently used Dutch selection throughout Europe is *Platanus × hispanica* ‘Alphen’s Globe’, which is much smaller than the parent species. Its broad leaves are also smaller, have 3–5 sharply serrated lobes, and are slightly hairy underneath. It develops a closed, regular spherical crown, shows vigorous growth, and its eventual height depends largely on the height of the graft. It is very resistant to hard, paved surfaces. It tolerates pruning and even shaping even in old age [23,32].

In addition to all these, without claiming to be complete, there are also a large number of other taxa available in the trade, such as the Hungarian varieties *Ginkgo biloba* ‘Globus’, *Tilia platyphyllos* ‘Pannonia’, and *Fraxinus ornus* ‘Villány’, the latter being the large sibling of the world-famous ‘Mecsek’. Also worth mentioning are *Acer campestre* ‘Anny’s Globe’, ‘Nanum’, *Gleditsia triacanthos* ‘Globosa’, *Liquidambar styraciflua* ‘Gum Ball’, *Prunus padus* ‘Nana’, *Tilia tomentosa* ‘Silver Globe’, or the Dutch varieties *Tilia cordata* ‘Monto’, ‘Lico’. These taxa occur in very small numbers in the country or are not yet established taxa.

In Budapest, among the priority tree rows maintained by the FŐKERT (nearly 40 thousand trees) and the trees along public transport routes (more than 100 thousand trees), the research includes nearly 5 thousand (4676) spherical crown trees in the 23 districts of Budapest, which are divided between 8 taxa.

### 2.3. Data Origin and Processing

For our work, we used the databases of the InfoGarden 2.1.2.1 (IG) GIS software. The software has been developed by Info-Garden Kft, Budapest, Hungary, since 2008, specifically for the map cadastral registration of public green spaces. The IG software uses a GIS-based, vector data model. The database runs on a postgreSQL database, and geometric calculations are performed by postGIS. The software establishes a dynamic connection between the map and background data during use. Each tree has a data sheet, in which the tree data can be accurately stored, such as species/variety, metric data, and health status, which are determined separately for the root, trunk, crown, and the tree as a whole. From the metric and health status data, the program calculates the value of the tree based on Radó’s tree value calculation [33,34]. Characteristics related to the tree’s environment can also be recorded on the data sheet, such as the size and material of the tree site and the presence of various utilities. The development and deterioration of the tree’s condition can be monitored based on the photo documentation. From the recorded data, we calculated leaf area, shadow projection, and canopy volume.

The correlation between measured trunk diameter and shade projection calculated from the measured canopy diameter was analyzed by correlation analysis. It is important to note that shading is considered the only ecosystem service in this study, not forgetting that this property has numerous urban ecological consequences.

### 2.4. The Tree Inspection and Assessment Methods

The trees’ measurement and evaluation in Budapest used the Hungarian Association for Tree Management’s (MFE) method and criteria [35]. The dendrological survey is the overall tree recording, where the dendrometric characteristics express the full tree height, trunk height, trunk circumference size, trunk diameter, and crown diameter in numbers.

A detailed examination is necessary because the habitat condition is often different from ideal, which affects the whole tree’s life chances. During the visual inspection, the general state of the roots, trunk, and crown is essential. We applied the EU conform method developed by MFE, which uses five parts and values:

A—Root system including roots and collars and the type and condition of tree’s plantation site;

B—Trunk condition;

C—Crown condition, including the crown base and the full crown;

D—Assessment of viability (Table 1).

The values of trees are based on MFE method as well:

Tree value = A × B × C × D × E × M

where A is nursery price of tree stocks (based on 2xi SF 12/14 quality), B is calculated age of the tree, C is tree’s protection and location within the municipality, D is condition of the crown base and crown, E is health and viability of the tree, and M is dendrological value of the tree species. In addition to the price of wood and age, each multiplier has several quality categories, each of which has a multiplier associated with it and is thus included in the formula [33,34].

In the context of urban tree assessment, three biometric variables (DBH, height, and crown width) are typically at the forefront of ecosystem services. They are used to predict other variables such as leaf area, canopy height, and tree biomass [36,37,38], which are necessary to estimate the ecological and economic benefits provided by trees. The model [39] was used to calculate crown volume (4/3 × π × r3), crown shadow projection area (π × cr2), and surface area (4π × cr2).

### 2.5. Statistical Analyses

The statistical analyses performed in the survey used the PAST 4.17 software package [40]. The comparison of medians (trunk diameter, shade projection area) was carried out using non-parametric tests (Mann–Whitney pairwise test for equal median). We made a correlation analysis to define the relationship between trunk diameter and shade projection area of studied taxa.

## 3. Results

### 3.1. Taxon Abundance, District Distribution, and Site Character

The most abundant species is *Robinia* in almost every district, and the total number of individuals in the studied area is 2928, which is almost twice as many as all other round trees combined (Table 2). Most of the spherical black locusts live in the 18th district, which is one of the outer districts of the city. The reason for their frequent use is probably a management decision because the district is located on the outskirts of the city, where the planting of small-crown trees is not particularly justified, especially not such a large-scale use of black locust. There are districts where the spherical trees are absent (District 5) or only appear in very small numbers (Districts 2, 7).

### 3.2. The Context (Relationship) of the Shade Projection Areas

**Figure 2:**
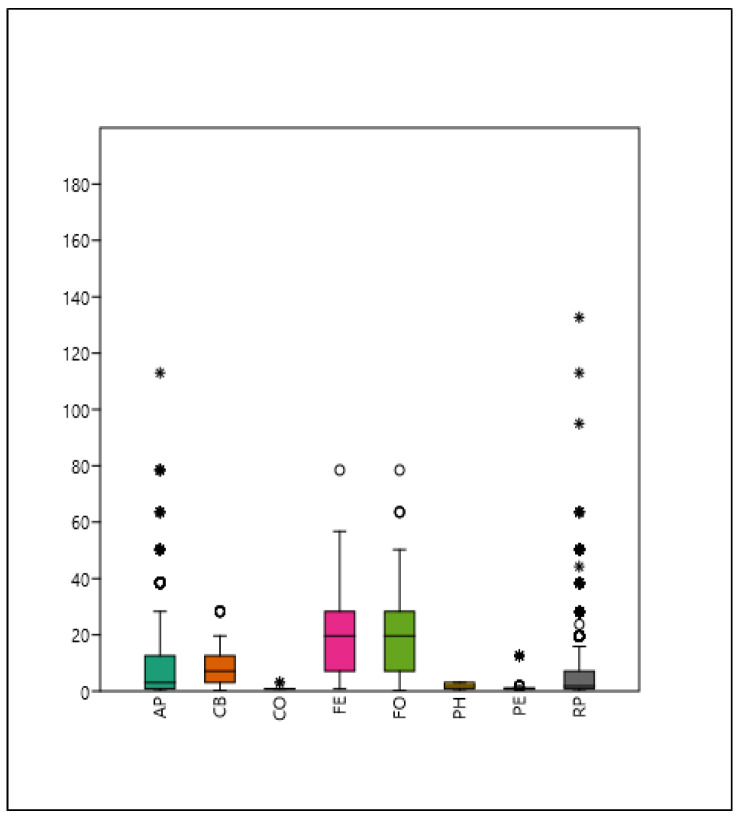
Boxplot of shade projection area (m^2^) for each examined taxon. Mean, median, 25–75% quartiles, non-outlier range, and outliers are denoted (see Table 2).

The median (FE–FO) of the two *Fraxinus* species is the largest and is approximately 89% identical. The shadow casts of the other taxa are significantly different from each other (Mann–Whitney pairwise test for equal median) (Figure 2).

### 3.3. The Relationship of the Trunk Diameter

**Figure 3:**
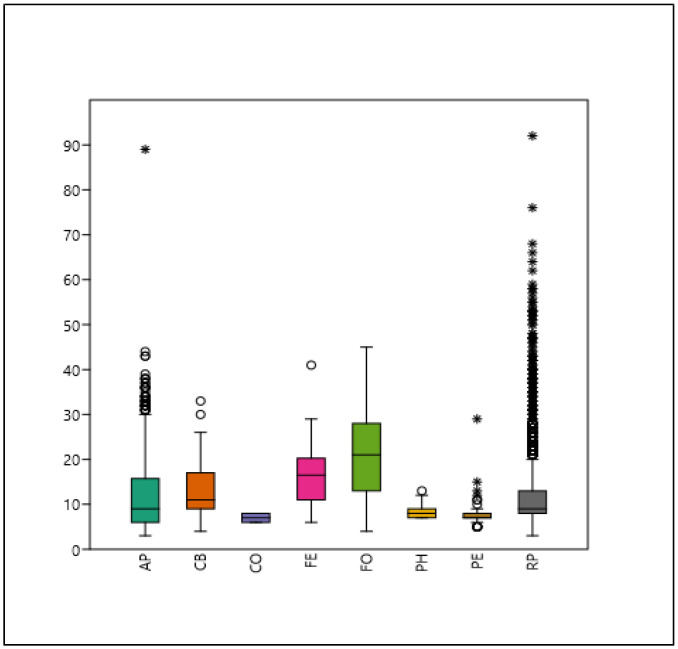
Boxplot of trunk diameter (cm) for each examined taxon. Mean, median, 25–75% quartiles, non-outlier range, and outliers are denoted (see Table 2).

There is no significant difference in the trunk diameter of the following species pairs: AP–PH; CO–PE; PH–PE. The largest diameters can be characterized for the FO and FE species (Figure 3).

### 3.4. The Relationship of the Trunk Diameter and Shade Projection Area

It can be stated that for almost all taxa, there is a close correlation between the trunk diameter and shadow projection (AP, CB, FE, FO, and PE). The negative correlation in the case of CE and PH is puzzling. The sign of the correlation coefficient (r) shows the direction of the relationship, and its magnitude (a number between 0 and 1) shows the closeness of the association and the strength of the relationship. However, it is wrong to claim that the correlation means a causal relationship (Table 3). It can be assumed that the negative relationship simply results from the small number of data (small sample).

### 3.5. Correlations Between Trees and Different Tree Locations

The trees live in different tree sites, of which the largest number of trees (3183 trees) is in category 2 (in green stripe, a continuous stripe covered with earth and/or turf of a given width) (Table 4). This is followed by category 1 (bed, the soil surface is also covered with some type of shrub, perennial, or annual plant) with 689 individuals, which is considered one of the most suitable urban habitats in terms of quality for linear, roadside developments.

**Figure 4:**
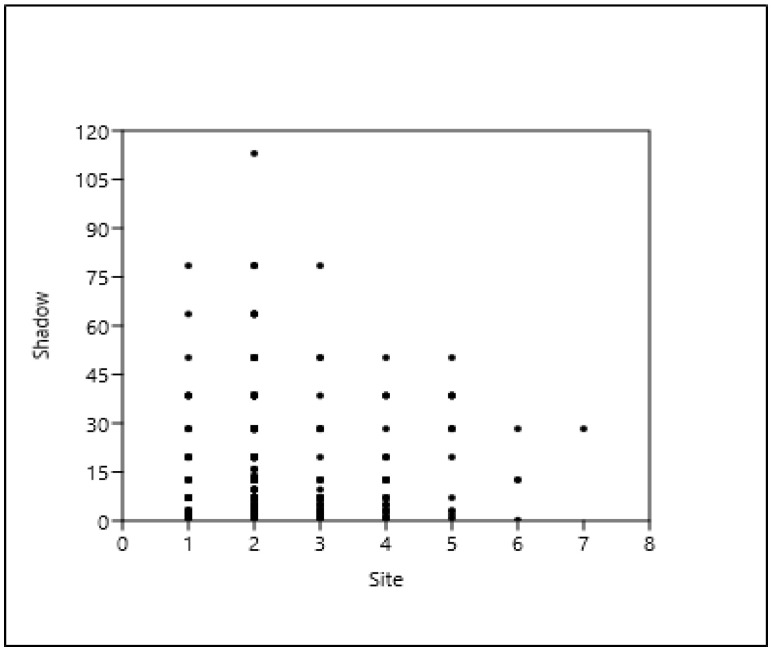
*Acer platanoides*, shade projection area (m^2^) (= shadow), and type of site (see Table 4).

**Figure 5:**
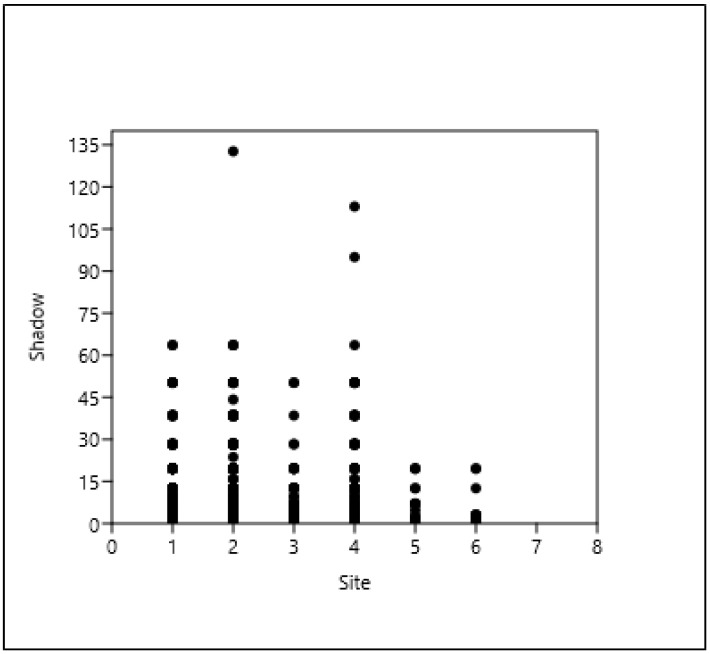
*Robinia pseudoacacia*, shade projection area (m^2^) (= shadow), and type of site. (see Table 4).

**Figure 6:**
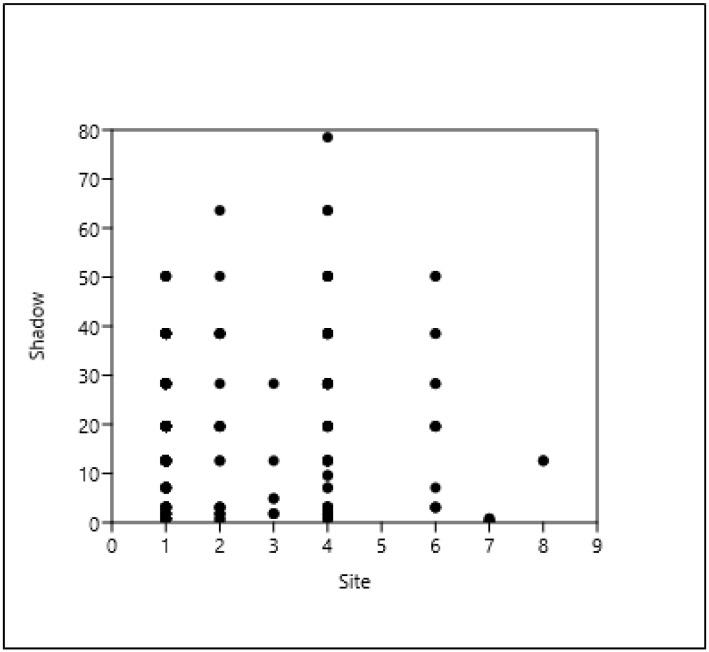
*Fraxinus ornus*, shade projection area (m^2^) (= shadow), and type of site (see Table 4).

Based on the results, the tree stands in the green belt (1–2–3) and the tree stands in the cover paving (4) have the most trees and the largest shadow projection for all three taxa examined here (AP, RP, FO), as shown in Figure 4, Figure 5 and Figure 6.

### 3.6. The Relationship Between the Distance of a Tree from a Building and the Shade Projection Area

**Figure 7:**
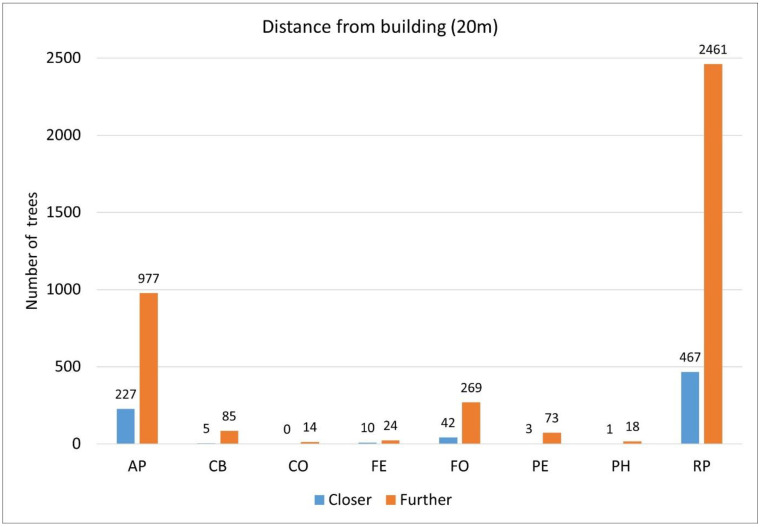
Distance from the building. Legend: closer (= number of individuals closer than 20 m), further (= number of individuals further than 20 m); see Table 2.

Based on the preliminary hypotheses, it can be expected that there is a negative correlation between the distance of the tree site from the house and the shadow projection, but according to the evaluations, no close correlation can be demonstrated based on the recorded data. There is no close correlation between the shadow projection (m^2^) and the distance from the building (<20 m) among the examined individuals, and, due to the insufficiency of the data (small sample size), only the analysis of AP, FO, and RP makes sense (Figure 7). The correlation between the shadow projection and the distance from the building (less than or greater than 20 m) for these taxa is 0.14204, 0.25132, and 0.0287, i.e., there is neither a close positive nor a close negative correlation (>0.8, <−0.8).

### 3.7. Age and Health of Trees and Their Correlation at Different Tree Sites

**Figure 8:**
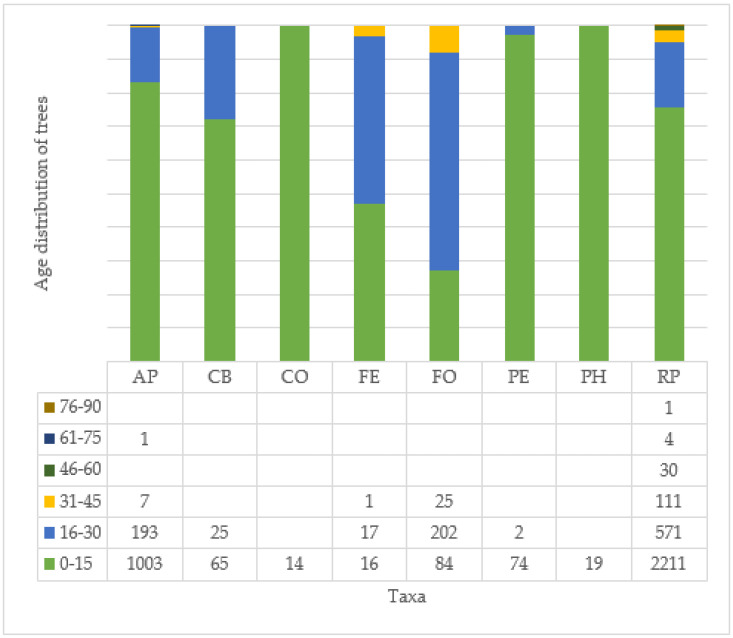
Age distribution of the whole recorded evaluated trees (see Table 2).

In almost all the spherical cultivars, the majority of the trees planted in the city are young (0–15 years), except in both taxa of the genus Fraxinus (FO, FE), where the majority of trees are between 16 and 30 years old. The large RP taxa have specimens in all categories, although more than 98% of the trees are less than 45 years old here too (Figure 8).

**Figure 9:**
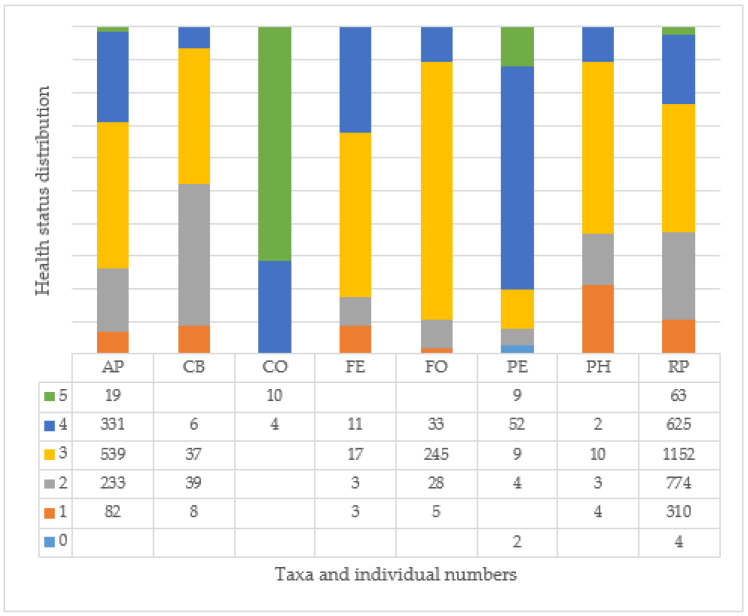
Health status distribution of the whole recorded tree cultivars (see Table 1 and Table 2).

Proportionately, the CO species has the highest number of healthy or good condition specimens, although there are few young trees in Budapest overall. The PE species is diverse, with all categories of trees, but with a higher proportion of trees in good and excellent health. The other species have a high proportion of trees in medium health (3). For the CB, PH, and RP varieties, the individuals studied seem to be more in medium or poorer health (Figure 9). For AP, CB, FE, FO, PH, and RP taxa, there are a certain number of individuals in poor health (2) and condemned (1) to be felled, compared to the number of individuals planted, and, for PH and RP, there are even individuals where only the empty tree site is recorded in the inventory. In the case of the pear trees examined, 1496 (32%) individuals are listed in health categories 1 and 2.

**Figure 10:**
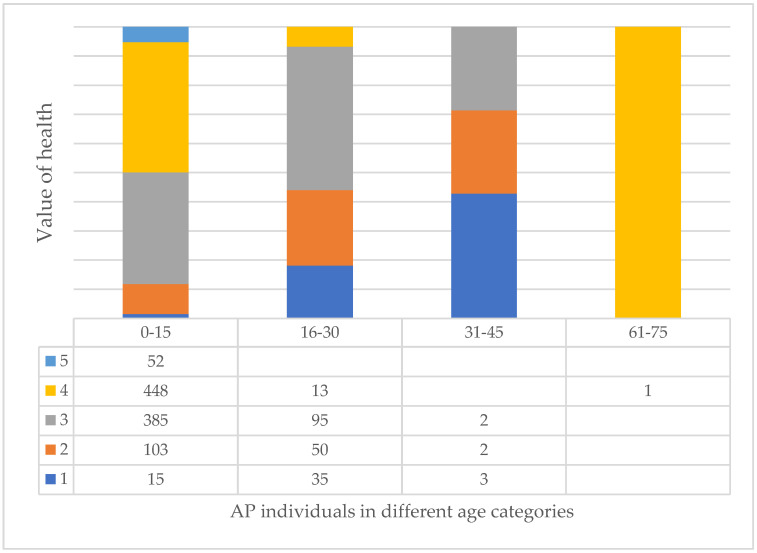
Relationship of value of health of trees and age categories in case of AP (see Table 1).

**Figure 11:**
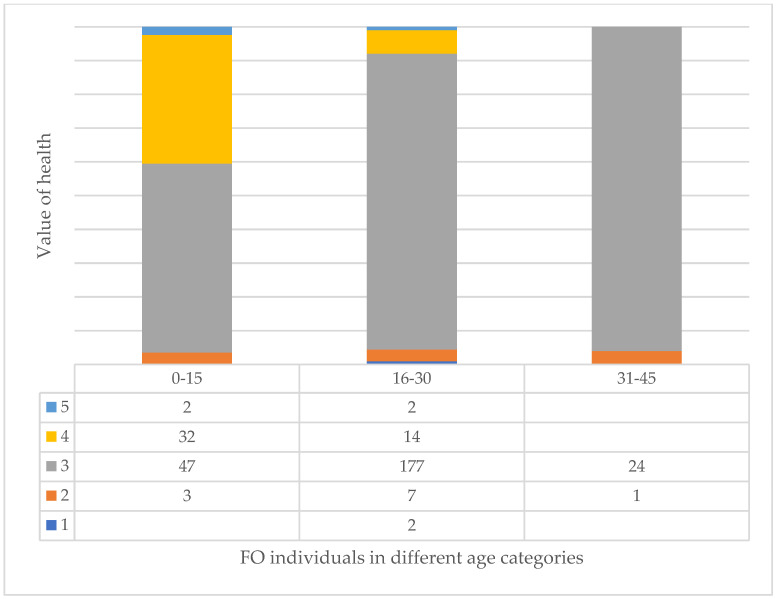
Relationship of value of health of trees and age categories in case of FO (see Table 1).

**Figure 12:**
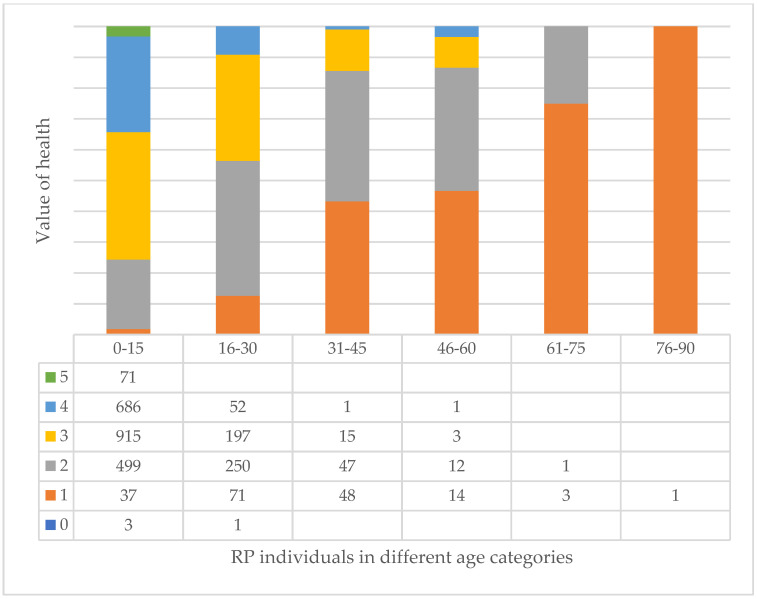
Relationship of value of health of trees and age categories in case of RP (see Table 1).

In fact, if we look at the three largest species, we can clearly see that the AP species improve with age (Figure 10), while the FO (Figure 11) and RP (Figure 12) species deteriorate significantly, especially the RP trees, where 48% of the 46–60 age class are already in a highly deteriorated state, i.e., recommended for felling.

**Figure 13:**
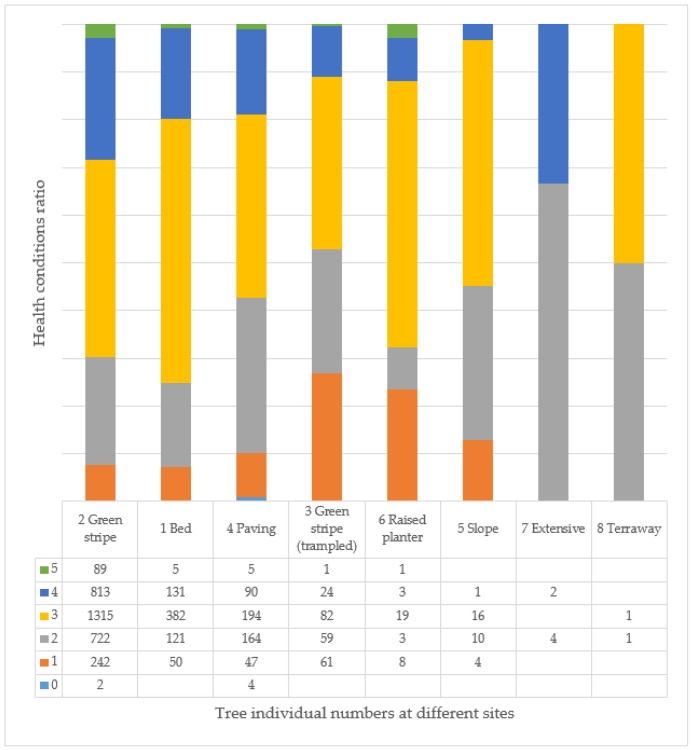
Condition of the examined trees at different tree sites (see Table 1 and Table 3).

**Figure 14:**
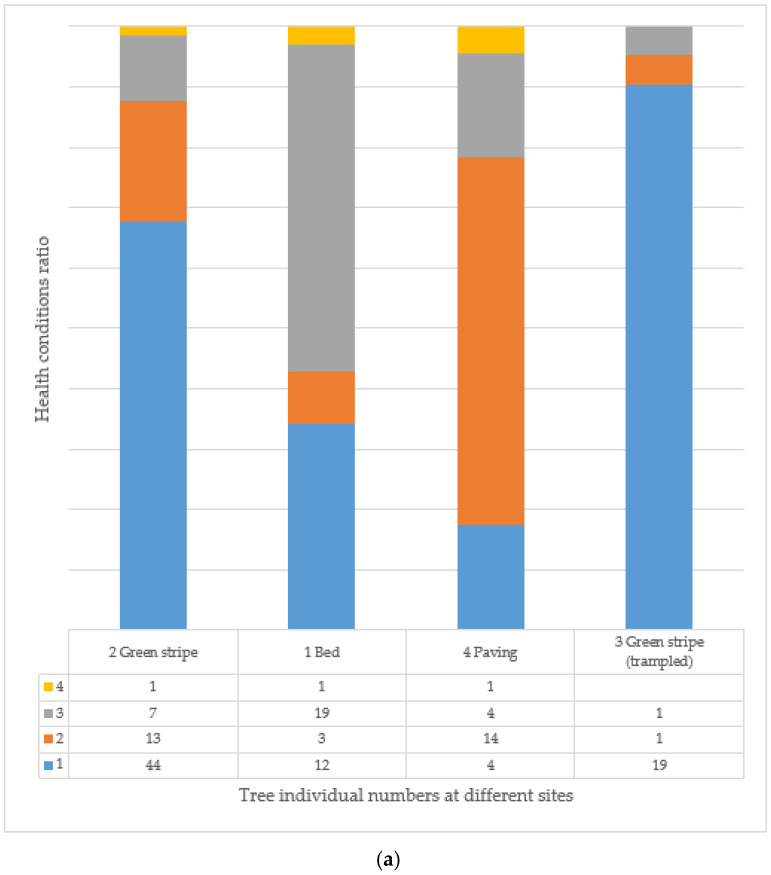

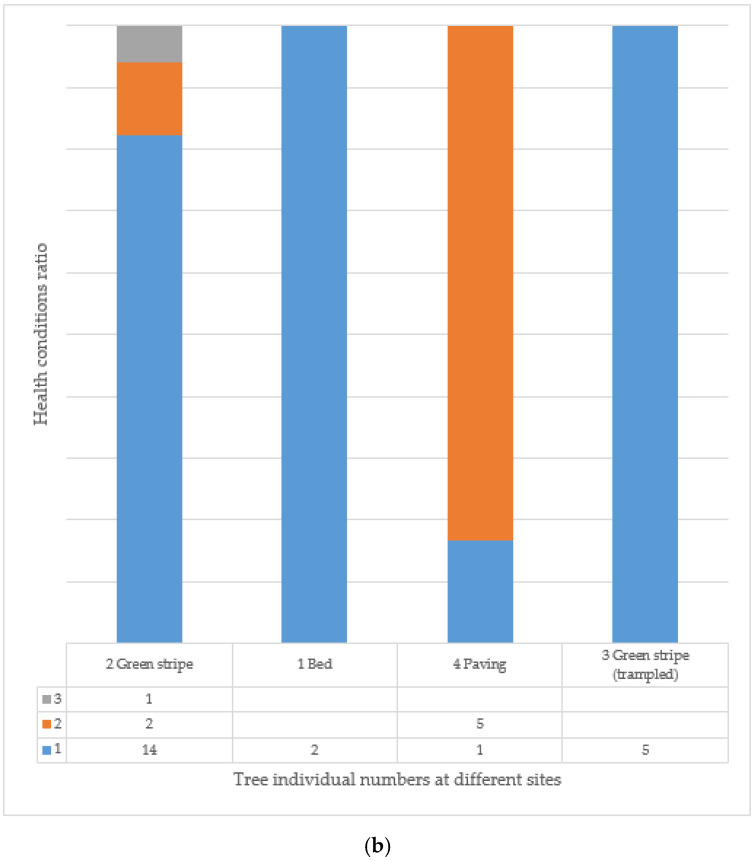
(**a**,**b**) The condition of the examined trees in different tree locations and age groups: (**a**) 31–45 years old, (**b**) 46–60 years old (see Table 1 and Table 3).

When evaluating all individuals, most individuals (3183) live in tree location type 2, followed by significantly fewer in tree locations 1 (689 trees) and 4 (504 trees). There are 227 trees in the dense green belt (3), and the other tree location categories (5–8) are negligible due to the small number of individuals (73 trees). There are only 101 trees with a health value of 5, of which 89 live in tree location 2 (Figure 13). When evaluated by age group, individuals with health status 4 still occur in the 31–45 age group in tree locations 1, 2, and 4 (Figure 14a), but in the 46–60 age group, trees can only be evaluated in health categories 1–3 (Figure 14b). In the trodden green stripe (trampled), in the type 3 tree site, 90% of the trees have a health value of 1, so they are recommended for felling, and the situation is even worse for trees older than this, as all individuals are recommended for felling. Since, in this age group and the older ones (61–75, 76–90), with one exception (1 AP tree in the 61–75 age group), there are only RP individuals; therefore, the use of the species should be considered from this point of view as well.

## 4. Discussion

In the relationships between age, trunk diameter, and crown projection, there is a positive correlation for almost all taxa, at least for the taxa (AP, CB, FE, FO and PE) that were planted in Budapest in larger numbers. The trunk of FO and FE thickens well and in light of this, the crown projection and shading ability also stand out among the taxa. For this reason, these taxa are the best in terms of ecosystem services (shade projection, microclimate), and their application is most justified. The greatest growth is expected for the FO taxon, especially if we also take into account that its urban lifespan can be considered good and it is not susceptible to the fungal disease causing ash dieback (*Hymenoscyphus fraxineus* syn. *Chalara fraxinea*), while FE unfortunately is [41,42,43]. For this reason, FO may be the largest ecosystem service provider among the examined spherical crown taxa.

The hypothesis that the proximity of the building clearly determines the size of the tree canopy and thus this segment of the ecosystem service with the shadow projection could not be supported by our studies. The reason for this is not that the restriction of the facade of buildings would have less of an effect on trees with spherical crowns or that it is not considered a negative environmental factor, but that the 20 m recorded in practice when recording cadastral data are not really interpretable in this case. It can be formulated as a suggestion that more detailed data recording is expedient in the case of nearby buildings, and thus crown distortions and asymmetries can be followed in the cadastral register even in the case of smaller crowns or young trees, which impose an additional burden on both maintenance and the later life of the trees.

Differences in age composition may indicate health conditions. In the case of varieties that we know are young breedings, we can actually talk about an introduction period, such as in the case of CO. In the case of PE, early crown deterioration can indeed lead to mainly young individuals throughout the city. Although PH and CB have old varieties, they appear in small numbers in the plantings. In the case of PH, it can be said that the basic species is still a common tree in the city, but old individuals are only beautiful in habitats and tree stands where the proportion of cover is minimal; instead, there is a green belt or green area around the trees. Without exception, young individuals of the plane tree spherical variety all live in grassy green belts, not in cover. The CB variety lives in various tree stands, mostly green belts, which are mainly grassy or covered with shrubs and herbs, but it can also be found in selected tree stands and covers in a really bad habitat. Preserving natural habitats, even in a green belt, e.g., creating herbaceous or shrub surfaces instead of compacted soil and lawns, promotes and improves the development of soil life and thus the condition of trees will also be better [42,44]. It is therefore no coincidence that in all the taxa examined, the best health conditions in the young category (0–15) are observed in individuals living in the green belt (types 4, 5). The environment and site specificity of the tree clearly determine the conditions for the development of the root system [45] and the development of the canopy [46]. Trees with weakened vigor can provide lower performance in the ecosystem service of urban green areas [47].

**Figure 15:**
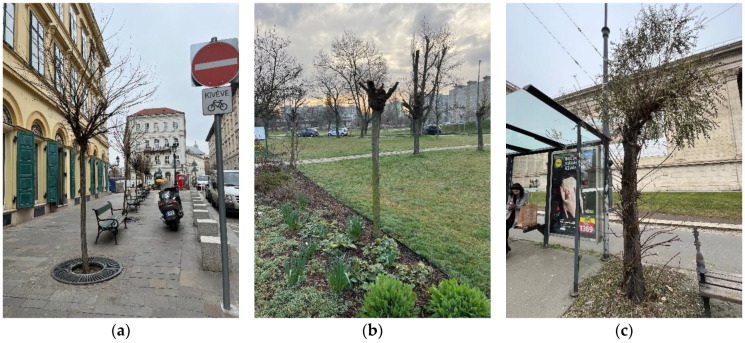
Black locust trees in Budapest, (**a**) young specimens in a square with newly restored medieval traces in district 6, (**b**) young specimen after crown trimming in district 4, (**c**) older tree in very poor health in district 14.

The health values of the whole tree are very diverse. The best general health values appear in the CO variety. The PE variety also has a higher level of good and excellent conditions, but we know that they mostly die young, so while they are young they are beautiful, compact, and have a closed spherical crown, but then they suddenly deteriorate [7]. If we look at the age group assessments, we can see that the health of most tree species weakens in old age. The most extreme example of this is RP, where individuals recommended for felling appear in the age group of 16–30. The reason for this is that the grafting site rots early [24], and a weaker storm can mean the breaking off of the crown branches and the loss of a large part of the crown (Figure 15). All this requires continuous replacement for urban forestry [48], which also does not serve an effective fight against the climate. The extinction of large trees has serious consequences [49,50,51,52,53,54,55], and it is impossible to compensate fairly, but the constant replacement of young, lower-performing trees also keeps the canopy cover of the city at zero, i.e., does not advance it. It is also worth noting here that in the case of RP, pruning to the bald head is very common, which further worsens the above. Although RP individuals play a major role in increasing the nutrient content of the soil [56,57], the ecosystem service of the above-ground parts lasts an extremely short time. Horváthová et al., 2021, showed that the initial costs (planting, maintenance) of short-lived trees are much higher than, for example, the shading benefit [58]. According to his research, this situation is reversed in the case of lifespans over 40 years. Small trees therefore do not achieve high ecosystem service efficiency [47], in which case even a shrub instead has more beneficial properties in the long term.

In addition, our studies also support that RP cannot be recommended for planting at all, neither in terms of maintenance difficulties nor ecosystem services. In addition, the taxa (CB, PH) where the health value is high (>30%) in relation to the planted individuals in health categories 2, 1, and 0 should also be given less importance in the future planning of public space trees. In the CB taxon, more than half (52%) of the individuals fall into this category, while it is 37% in the PH taxon and 38% in the case of RP.

Among the taxa examined, those where more than 70% of the planted individuals fall into the health categories 3, 4, and 5 can still be recommended for urban plantings, such as AP, CO, FE, FO, and PE. Among them, FO can be clearly highlighted with 89% and PE with 92%, as well as the already mentioned CO with 100%; however, among the highlighted ones, in terms of species characteristics, durability of the crown structure, tolerance to diseases and pests, and long foliage retention [10,59], FO clearly comes out victorious.

From the perspective of tree health and long-term provision of ecosystem services, it would be worthwhile to involve local communities in urban green space management processes in environmental education practices, as several researchers have formulated [60]. In addition to the evaluation of ecosystem services, the monetization approach can also assign a financial value to municipal assets based on market prices and pruning, maintenance, and replacement costs, etc. [4,61], which can potentially support urban forestry [62]. Thus, in the case of the FŐKERT, it is worth avoiding short-lived, poorly healthy taxa, which also attract high maintenance (e.g., pruning) costs; instead, the planting of healthy, long-lasting, larger-crown taxa should be encouraged, which are associated with fewer monetization costs.

## Figures and Tables

**Table 1:** Assessment of the viability of the tree and its organs [35].

Root Condition		Trunk Condition	
Evaluation	Grade	Evaluation	Grade
intact root necks, optimal growing conditions	5	intact	5
intact root necks, acceptable growing conditions	4	superficial injuries	4
less damage to root crowns, acceptable growing conditions	3	some superficial wounds and aging	3
heavy visible yellowing, unfavorable growing conditions	2	large surface wounds, serious decay	2
severe damage, very poor site	1	progressive decay, necrosis	1
dead roots, empty tree site	0	empty tree site	0
**Crown Condition**		**Health and Viability of the Tree**	
**Evaluation**	**Grade**	**Evaluation**	**Grade**
crown shape intact, foliage loss (1–10%)	5	excellent condition	5
foliage loss (11–25%)	4	with intervention near maximum age	4
significant defoliation (26–50%)	3	to be replaced before maximum age	3
severe crown damage ((over 50%)	2	to be replaced within 10 years	2
dead crown, total defoliation	1	to be replaced urgently	1
empty tree site	0	empty tree site	0

**Table 2:** The occurrence of taxa in different districts of Budapest.

Districts	*Robinia pseudoacacia* ‘Umraculifera’	*Acer platanoides* ‘Globosum’	*Fraxinus ornus* ‘Mecsek’	*Fraxinus excelsior* ‘Nana’	*Celtis occidentalis* ‘Globosa’	*Prunus* × *eminens* ‘Umbraculifera’	*Platanus* × *hispanica* ‘Alphen’s Globe’	*Catalpa bignonioides* ‘Nana’
	RP	AP	FO	FE	CO	PE	PH	CB
**I.**		**2**	**14**					
**II.**	**1**		**1**			**1**		
**III.**	**24**	**12**		**4**	**10**			**8**
IV.	318	2	1					
V.								
VI.	145		2					
VII.			1					
VIII:	238	11	10		4			
IX.	50	9						
X.	305	109	9	3		30		
**XI.**	**43**	**21**	**169**	**1**		**16**		**8**
**XII.**	**105**	**10**	**64**					
XIII.	64	12	14	7				
XIV.	84	7	7	2		17		7
XV.	144	100						3
XVI.	74	392		1				1
XVII.	190	198				12	19	10
XVIII.	699	106	5	6				4
XIX.	189	27	11					
XX.	62	19						
XXI.	137	47		3				
**XXII.**	**5**	**58**	**3**					**35**
XXIII.	51	62		7				14
Total	2928	1204	311	34	14	76	19	90

Note: The districts in bold are located on the Buda side of the city, where the climate of the Buda hills may provide more favorable conditions. The location of the districts is shown in Figure 1.

**Table 3:** Correlation coefficients (r [−1, 1]) between trunk diameter and shade projection area (bold: strong positive correlation r ≥ 0.7; species list is in alphabetical order).

***Acer platanoides***	**AP**	**0.74**
***Catalpa bignonioides***	**CB**	**0.87**
*Celtis occidentalis*	CO	−0.47
***Fraxinus excelsior***	**FE**	**0.88**
***Fraxinus ornus***	**FO**	**0.78**
*Platanus × hispanica*	PH	−0.11
***Prunus × eminens***	**PE**	**0.76**
*Robinia pseudoacacia*	RP	0.68

**Table 4:** Number of trees examined in different tree sites.

NO	Types	Descriptions	Number of Individuals
1	Bed	the soil surface is also covered with some type of shrub, perennial, or annual plant	689
2	Green stripe	in the tree line, a continuous strip covered with earth and/or turf of a given width	3183
3	Green stripe (rampled)	the soil is compacted, which is usually caused by vehicles parked in the wooded area	227
4	Paving	the tree site is surrounded by some kind of closed covering, for example, concrete, asphalt, paving stones	504
5	Slope	tree site in a sloping area, e.g., it is on the bank of a ditch, on the side of an overpass, on a hillside	31
6	Raised planter	the wood site has a raised, concrete, brick, or stone border and the ground surface in the wood site is higher than the surface or pavement surrounding the wood site	34
7	Extensive	the environment of the tree is not well kept, usually outside, neglected areas on the side of the road	6
8	Terraway	the floor of the enclosure is covered by a water-permeable covering called Terraway	2

## Data Availability

Data no available, legal restriction, data connected to FŐKERT.
